# Optimization of Y-90 Radioembolization Imaging for Post-Treatment Dosimetry on a Long Axial Field-of-View PET/CT Scanner

**DOI:** 10.3390/diagnostics13223418

**Published:** 2023-11-09

**Authors:** Pia M. Linder, Wenhong Lan, Nils F. Trautwein, Julia Brosch-Lenz, Sebastian von Beschwitz, Jürgen Kupferschläger, Gerald Reischl, Gerd Grözinger, Helmut Dittmann, Christian la Fougère, Fabian P. Schmidt

**Affiliations:** 1 Department of Nuclear Medicine and Clinical Molecular Imaging, University Hospital Tuebingen, 72076 Tuebingen, Germany; pia.linder@med.uni-tuebingen.de (P.M.L.); wenhong.lan@med.uni-tuebingen.de (W.L.); sebastian.beschwitz@med.uni-tuebingen.de (S.v.B.); christian.lafougere@med.uni-tuebingen.de (C.l.F.); helmut.dittmann@med.uni-tuebingen.de (H.D.); 2 Department of Nuclear Medicine, Klinikum Rechts der Isar, Technical University Munich, 81675 Munich, Germany; 3 Werner Siemens Imaging Center, Department of Preclinical Imaging and Radiopharmacy, Eberhard-Karls University Tuebingen, 72074 Tuebingen, Germany; gerald.reischl@med.uni-tuebingen.de; 4 Cluster of Excellence iFIT (EXC 2180) “Image Guided and Functionally Instructed Tumor Therapies”, University of Tuebingen, 72074 Tuebingen, Germany; 5 Department for Diagnostic and Interventional Radiology, University Hospital Tuebingen, 72076 Tuebingen, Germany; gerd.groezinger@med.uni-tuebingen.de

**Keywords:** PET/CT, SIRT, radioembolization, Y90, total body PET, LAFOV

## Abstract

Background: PET imaging after yttrium-90 (Y-90) radioembolization is challenging because of the low positron fraction of Y-90 (32 × 10^−6^). The resulting low number of events can be compensated by the high sensitivity of long axial field-of-view (LAFOV) PET/CT scanners. Nevertheless, the reduced event statistics require optimization of the imaging protocol to achieve high image quality (IQ) and quantification accuracy sufficient for post-treatment dosimetry. Methods: Two phantoms (NEMA IEC and AbdoMan phantoms, mimicking human liver) filled with Y-90 and a 4:1 sphere (tumor)-to-background ratio were scanned for 24 h with the Biograph Vision Quadra (Siemens Healthineers). Eight patients were scanned after Y-90 radioembolization (1.3–4.7 GBq) using the optimized protocol (obtained by phantom studies). The IQ, contrast recovery coefficients (CRCs) and noise were evaluated for their limited and full acceptance angles, different rebinned scan durations, numbers of iterations and post-reconstruction filters. The s-value-based absorbed doses were calculated to assess their suitability for dosimetry. Results: The phantom studies demonstrate that two iterations, five subsets and a 4 mm Gaussian filter provide a reasonable compromise between a high CRC and low noise. For a 20 min scan duration, an adequate CRC of 56% (vs. 24 h: 62%, 20 mm sphere) was obtained, and the noise was reduced by a factor of 1.4, from 40% to 29%, using the full acceptance angle. The patient scan results were consistent with those from the phantom studies, and the impacts on the absorbed doses were negligible for all of the studied parameter sets, as the maximum percentage difference was −3.89%. Conclusions: With 2i5s, a 4 mm filter and a scan duration of 20 min, IQ and quantification accuracy that are suitable for post-treatment dosimetry of Y-90 radioembolization can be achieved.

## 1. Introduction

Radioembolization, also known as selective internal radiotherapy (SIRT), is a locoregional treatment option for hepatocellular carcinoma (HCC) and liver metastasis originating from primary tumors, such as neuroendocrine tumors (NET) and colorectal carcinoma (CRC) [1,2]. For radioembolization, microspheres loaded with the beta-minus-emitter yttrium-90 (Y-90) are injected into the intrahepatic arteries to achieve a high local dose deposition directly to the tumor or metastases [3].

Before treatment, technetium-99m-labeled macroaggregated albumin (Tc-99m-MAA), serving as a surrogate for the Y-90 spheres, is injected, and a Tc-99m-MAA single-photon emission (SPECT)/computed tomography (CT) scan is performed to assess extrahepatic shunting, plan the treatment activity and derive the expected absorbed dose for the tumor and healthy liver parenchyma, in order to ensure safe treatment [4].

Potential deviations in the activity distribution between the planning and treatment make treatment verification essential. Post-treatment Y-90 Bremsstrahlung SPECT/CT is the standard method for this, despite its limited spatial resolution and lack of intrinsic activity quantification [5,6].

Y-90 positron emission tomography (PET) imaging has gained popularity for post-therapy imaging of radioembolization, due to its high quantification accuracy for dosimetric purposes [5,6,7]. However, Y-90 PET imaging is severely limited by the low branching ratio of only 32 × 10^−6^ [8] for internal pair production, resulting in a low signal-to-noise ratio (SNR) and the need for long scan times. 

The high sensitivity of long axial field-of-view (LAFOV) scanners allows Y-90 PET imaging with improved image quality within moderate scan times [9]. This enables routine post-therapeutic Y-90 PET imaging with accurate quantification and localization of Y-90 spheres [10], which can be used for individual patient post-therapeutic dosimetry, as advised by Levillain et al. [11] and the European Association of Nuclear Medicine guidelines [12].

As Y-90 PET imaging is currently not established as a standard in clinical routines, there is no harmonization or standardization of image reconstruction parameters or scan protocols; however, evaluations for standard-field-of-view (SAFOV) systems have already been performed [9,13,14,15,16].

The aim of this study was to evaluate and optimize the scan protocol and reconstruction parameters for Y-90 PET imaging using the Biograph Vision Quadra LAFOV PET/CT scanner (Siemens Healthineers, Knoxville, TN, USA). 

Firstly, evaluations were performed using the National Electrical Manufacturers Association (NEMA) International Electrotechnical Commission (IEC) phantom guidelines for standardized image quality performance assessment. Secondly, comprehensive evaluations were performed to optimize the scan protocol and reconstruction parameters using the AbdoMan phantom [17]. This mimics a human liver with tumors, and therefore better represents the geometry and activity distribution in radioembolization patients. Thirdly, an evaluation was performed with a cohort of *n* = 8 radioembolization patients with different hepatic malignancies (HCC, metastases from NET or CRC) to assess the transferability of the scan and reconstruction protocol optimization obtained from the phantom experiments, as well as the suitability for dosimetry. 

## 2. Materials and Methods

### 2.1. Biograph Vision Quadra PET/CT Scanner

All of the measurements were performed on a Biograph Vision Quadra (Siemens Healthineers, Knoxville, TN, USA) PET/CT system with an axial FOV of 106 cm using software version VR10. All possible lines of response (LORs), i.e., with a maximum ring difference (MRD) of 322 crystal rings (52° acceptance angle) were recorded. In the image reconstruction process of software version VR10, the MRD was limited to 85 (18° acceptance angle). The MRD85 and MRD322 reconstructions are also referred to as high-sensitivity (HS) and ultra-high-sensitivity (UHS) modes, with reported NEMA NU 2-2018 sensitivities of 83 cps/kBq and 176 cps/kBq, respectively [18].

### 2.2. Image Reconstruction and Analysis Software

In order to enable the comparison of the HS and UHS modes, all of the datasets were reconstructed with the e7 tools (Siemens Healthineers, Knoxville, TN, USA) using software version VR20, which supports both sensitivity modes. All of the images were reconstructed into a 440 × 440 × 645 matrix with an isotropic voxel size of 1.65 mm using an ordinary Poisson-ordered subsets expectation maximization (OP-OSEM) algorithm with point spread function (PSF) modeling and time-of-flight (TOF) information. Attenuation correction for all phantom scans was based on a prior standard diagnostic CT scan (210 mAs reference current, 120 kVp). Next to the two sensitivity modes, different iterations (i) and subsets (s) for the iterative image reconstruction ranging from 1i5s to 5i5s were evaluated, and post-reconstruction Gaussian image filters of 2, 4, 6 and 8 mm full-width-at-half-maximum (FWHM) were applied. The image data were analyzed with AMIDE version 1.0.4 [15] for the phantom studies, and the Affinity Viewer^®^ (Hermes Medical Solutions, Stockholm, Sweden) was additionally used for the patient study.

### 2.3. IEC Phantom Measurements and Analysis of IQ, Contrast Recovery, Image Noise and Lung Residual Error

A standard IEC phantom with six fillable sphere inserts (diameters 10, 13, 17, 22, 28 and 37 mm) and a lung insert were used, according to the NEMA NU 2-2018 standard [19]. The phantom was first flushed with diluted hydrochloric acid (0.01 mol/L) containing non-radioactive Y-89 ions (2.5 mg/L) to saturate potential binding sites inside the phantom walls to prevent Y-90 binding to the phantom walls. Then, the phantom background was filled with an aqueous solution of 20% diethylenetriaminepentaacetic acid (DTPA), buffered by sodium acetate, and Y-90 chloride with an activity concentration of 270 kBq/mL and the spheres were filled with an activity concentration of 1020 kBq/mL, resulting in a SBR of 3.78:1 and a total activity of 2.6 GBq at the scan start. The phantom was placed with the smallest sphere centered in the axial FOV, while the lung insert was centered in the transaxial FOV. The scan duration for the reference scan in terms of best image quality (IQ) and quantification accuracy was 24 h. Emission scans of 30 min and 20 min acquisition times were obtained by rebinning of the listmode data.

Image reconstruction was performed with 2i5s, either with a 2 mm FWHM Gaussian filter or without filtering, to what we refer to as a 0 mm filter. The contrast recovery was assessed via the contrast recovery coefficient (CRC), adapted from the NEMA NU 2-2018 [19] standard. The CRC was calculated according to the following equation:

(1) $${CRC}_{i}=\frac{\frac{C_{i}}{C_{BG}}-1}{\frac{A_{i}}{A_{BG}}-1}\times 100\%$$

where C_i_ is the measured mean activity concentration in sphere i, C_BG_ the measured mean activity concentration in the background and A_i_ and A_BG_ are the real activity concentrations in the sphere and background, respectively. The C_i_ was determined from spherical volumes of interest (VOIs) with diameters matching the respective sphere size (10, 13, 17, 22, 28 and 37 mm) of the IEC phantom spheres, and the C_BG_ was determined from a box-shaped VOI of 150 mm × 15 mm × 170 mm.

The image noise was assessed using the coefficient of variation (CV), a widely used parameter for comparison of the noise performance of PET scanners [20,21]. The CV was calculated according to the following equation:

(2) $${CV}_{BG}=\frac{{SD}_{BG}}{C_{BG}}\times 100\%$$

where SD_BG_ is the standard deviation of voxel values in the background VOI. The lung residual error was calculated according to the following equation:

(3) $$Lung\;Residual\;Error=\frac{C_{Lung}}{C_{BG}}\times 100\%$$

where C_Lung_ is the mean activity concentration determined from a cylindrical VOI with a diameter of 30 mm and a depth of 127 mm placed inside the lung insert.

### 2.4. AbdoMan Phantom Measurements and Analysis of IQ, Contrast Recovery, Image Noise and Lung Residual Error

Measurements with the AbdoMan phantom were performed to better approximate the clinical performance of the scanner for Y-90 scans of radioembolization patients. This anthropomorphic 3D-printed phantom mimics the human abdomen, especially the human liver, in regards to the geometry, tumor location, attenuation characteristics and liver volume. It is provided with different fillable inserts that represent tumor lesions [17].

Four fillable spherical inserts with diameters of 20, 30, 40 and 50 mm were used to imitate multiple tumor lesions of different sizes inside the AbdoMan phantom. The phantom liver background was, analogous to the IEC phantom, filled with an aqueous solution of buffered (sodium acetate) DTPA and Y-90 chloride. The activity concentration in the background was 299 kBq/mL and the spheres were filled with an activity concentration of 1103 MBq/mL, resulting in an SBR of 3.69:1 and a total activity of 635 MBq at the scan start. The phantom was axially and transaxially centered in the FOV, and a 24 h reference scan was performed with subsequent listmode data rebinning to 45 min, 30 min and 20 min. The impact of the sensitivity mode, number of iterations (1i5s to 5i5s), filter size (0, 2, 4, 6, 8 mm FWHM) and scan time (24 h, 45 min, 30 min, 20 min) on IQ, contrast recovery and image noise were evaluated analogous to the IEC phantom analysis. The activity concentrations for the spheres were obtained via spherical VOIs with 20, 30, 40 and 50 mm, corresponding to the respective sphere sizes. The activity concentration for the background was derived from a VOI 50 mm in diameter placed in the liver background.

### 2.5. Patient Study and Analysis of IQ, Image Noise and Accuracy for Dosimetry

Eight patients with HCC, NET or CRC who underwent radioembolization therapy with Y-90 microspheres were included in this study. The study was conducted in accordance with the Declaration of Helsinki, and was approved by the ethics committee of the Faculty of Medicine, University of Tuebingen. Informed consent was obtained from all of the patients involved. Four of them were treated with resin spheres (SIR-Spheres, Sirtex SIR-Spheres Pty Ltd., North Sydney, Australia) and four were treated with glass spheres (TheraSphere, Boston Scientific, Marlborough, MA, USA). The total treatment activity ranged from 1285 MBq to 4659 MBq, and the injections were administered either via one or two catheter positions. Individual details on the patient cohort are depicted in Table 1.

The treatment planning for all of the patients was based on Tc-99m-MAA SPECT/CT scans using Affinity Viewer^®^ (Hermes Medical Solutions, Stockholm, Sweden), and the treatment activity calculation was based on the partition model [22]. All of the patients underwent a 30 min post-therapy scan on the Biograph Vision Quadra (Siemens Healthineers, Knoxville, TN, USA) with either a diagnostic (120 kVp, 210 mAs) or low-dose CT (140 kVp, <30 mAs) used for attenuation correction. Image reconstruction was performed in UHS mode. In order to evaluate the impact of a shorter scan time, the listmode data were rebinned to 20 min. The impact of the number of iterations (2i5s to 4i5s), filter size (2, 4, 6, 8 mm FWHM) and scan time on IQ and image noise was evaluated. The CV was determined using a spherical VOI 30 mm in diameter in the treated liver background.

A simplified dosimetry approach was chosen to evaluate the impact of the different acquisition and reconstruction parameters on the liver- and tumor-absorbed doses. Spherical VOIs with a diameter of 30 mm were placed in tumor areas and healthy liver tissue within the treated segment or treated liver lobe. The VOI statistics were extracted and the time-integrated activity (TIA) per VOI was calculated by dividing the decay-corrected total activity per VOI to the time point of Y-90 microsphere administration by the decay constant λ of Y-90. S-value-based dosimetry [23] was performed with IDAC spheres using the open-source dosimetry software IDAC-DOSE 2.1 [24]. The isotope selection was set to Y-90, and the material was set to liver tissue (density of 1.05 g/cm). The TIA per VOI was used in combination with the VOI volume to calculate the absorbed doses. Although this approach does not yield the actual mean healthy liver- or tumor-absorbed doses (ADs), it provides further insight on the impact of image acquisition and reconstruction on the absorbed dose. The percentage differences were calculated for the ADs between the different images using the 30 min, 2i5s and 4 mm filter image as a reference.

Various diseases show different tumor sizes and metastases, but also lead to differences in vascularization of the tumors affecting the tumoral to non-tumoral ratio (TNR). Therefore, to visually compare and evaluate the image quality, images of three exemplary patients with several tumor entities are presented in detail.

## 3. Results

### 3.1. IEC Phantom

**Figure 1:**
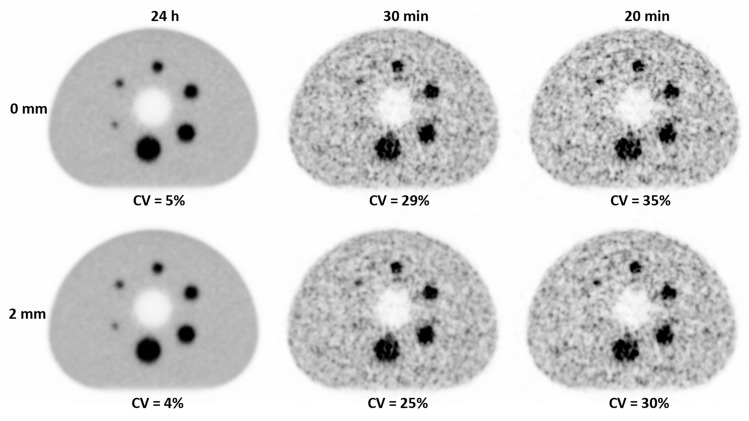
Transaxial views of the IEC phantom in UHS mode and 2i5s with CV values for different acquisition times (24 h, 30 min, 20 min), unfiltered (0 mm) and filtered (2 mm).

For shorter acquisition times, the noise was increasing, which corresponded with the respective CV values, e.g., increasing from 4% for 24 h to 25% for 30 min with the 2 mm filter (Figure 1). 

Furthermore, the 2 mm filter decreased the noise from 29% to 25% (30 min), and from 35% to 30% (20 min). 

Visualization of the smallest sphere was improved in the filtered images. However, it was not clearly visible for the 20 min acquisition. The CRCs remained stable for all sphere sizes and filters for the 20 min scan in comparison to the 24 h acquisition (Table 2).

The lung residual errors were 14% (24 h) and 12% (both, 30 min, 20 min), and were independent of filtering.

### 3.2. AbdoMan Phantom

**Figure 2:**
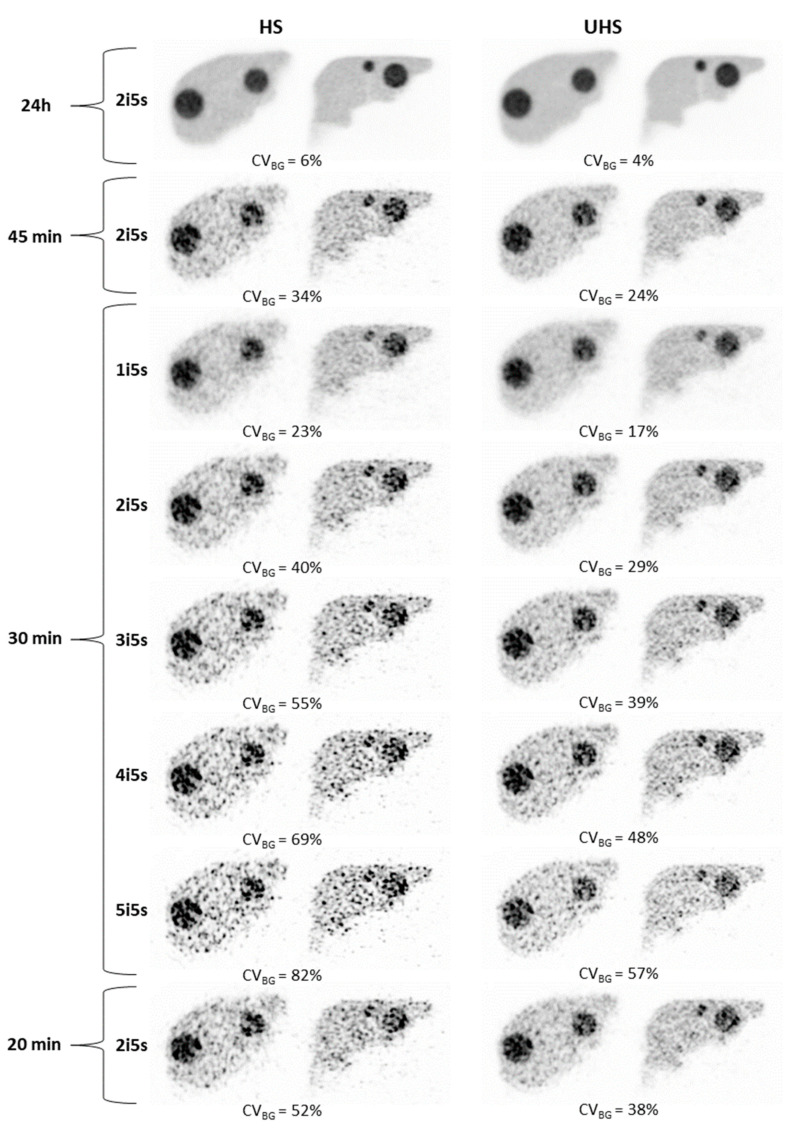
Transaxial and sagittal views of the AbdoMan phantom with CV values for both sensitivity modes and different numbers of iterations and acquisition times with 2 mm filter.

In correspondence to the IEC phantom results, the AbdoMan phantom image noise increased for shorter acquisition times, e.g., for UHS mode, 2i5s from 4% (24 h) to 29% (30 min) (Figure 2). In general, the images reconstructed in the UHS mode showed lower CV values compared to HS mode reconstruction, with the difference between both modes increasing towards shorter acquisition times, e.g., for 2i5s and 30 min, the CVs were 29% (UHS) and 40% (HS). The image noise increased with higher numbers of iterations, e.g., UHS mode, 30 min resulted in an increase in the CVs from 17% (1i5s) to 29% (2i5s) and 57% (5i5s).

**Figure 3:**
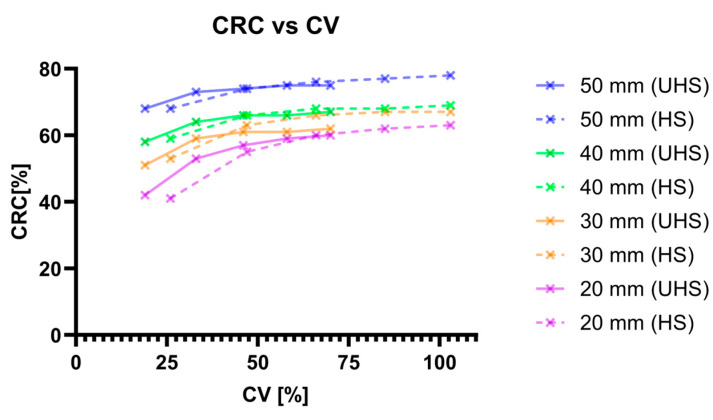
AbdoMan phantom CRC and CV for a 30 min acquisition time, different sphere sizes, both sensitivity modes and different numbers of iterations (no filter). Each point represents one iteration, and the number of iterations increases from left to right.

Next to the increase in noise for a higher number of iterations, the contrast recovery was improved (Figure 3).

Towards a higher number of iterations, the improvement in contrast recovery saturated, e.g., UHS mode, for 30 min and a 20 mm sphere the CRC values were 42%, 53%, 57%, 59% and 60% for 1i5s, 2i5s, 3i5s, 4i5s and 5i5s, respectively. The CRC was not improved beyond three iterations; however, the image noise further increased, e.g., UHS mode, for 30 min and a 50 mm sphere, the CRCs were 74% (3i5s) and 75% (5i5s) and the CVs were 46% (3i5s) and 70% (5i5s). The CRC values were comparable for the HS and UHS modes, e.g., 2i5s, 30 min, 40 mm sphere 66% (HS) and 64% (UHS), while the noise increased by a factor 1.4 with CVs of 47% (HS) and 33% (UHS). The absolute increase in noise with increasing numbers of iterations was in general higher for the HS mode in comparison to the UHS mode. The respective CRC values for 2i5s and 3i5s in UHS mode were comparable, e.g., 53% and 57% (20 mm sphere), 59% and 61% (30 mm), 64% and 66% (40 mm), 73% and 74% (50 mm); however, the CV values increased from 33% (2i5s) to 46% (3i5s). Based on these evaluations UHS mode reconstruction with 2i5s was determined to be optimal, as it offered a reasonable high contrast recovery and low noise; thus, it was used for the following evaluations.

**Figure 4:**
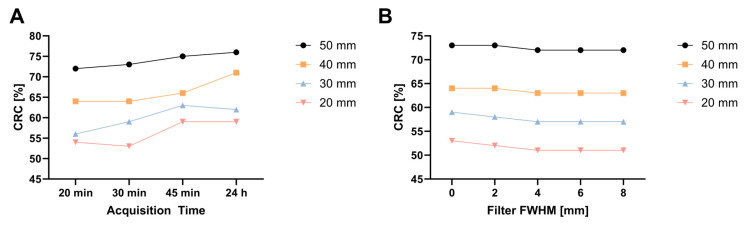
(**A**) AbdoMan phantom CRCs for different sphere sizes and acquisition times (UHS, 2i5s, no filter); (**B**) CRCs for different sphere sizes and filter (UHS, 2i5s, 30 min).

It was possible to maintain a high contrast recovery for all sphere sizes down to 20 min in comparison to the reference measurement with 24 h (Figure 4A), e.g., for the 30 mm sphere, the CRC values were 56% (20 min), 59% (30 min), 63% (45 min) and 62% (24 h).

After optimum sensitivity mode, the numbers of iterations and acquisition times were determined, and the impact of the filter size was evaluated. A negligible impact of the filter size on the contrast recovery was observed (Supplementary Materials Table S1), e.g., for the 20 mm sphere, the CRCs were 53% (0 mm filter), 51% (4 mm) and 51% (8 mm). The image noise decreased with larger filter sizes, shown by CV values of 33%, 29%, 24%, 23% and 23% for the 0, 2, 4, 6 and 8 mm filters, respectively. 

### 3.3. Patient Study Image Noise and Dosimetry

In accordance to the phantom study, the image noise increased in the patient study with increasing numbers of iterations (Table 3), e.g., for 30 min, 4 mm filter the CV values were 29 ± 9%, 34 ± 10% and 39 ± 11%, for 2i5s, 3i5s and 4i5s, respectively.

An increase in the filter size helped to mitigate the image noise; however, for a filter size larger than 4 mm, no further improvement was observed, e.g., for 30 min and 2i5s, the CV values were 34 ± 8%, 32 ± 8%, 29 ± 9% and 29 ± 9% for 0, 2, 4 and 6 mm filter sizes, respectively. A reduction in the scan time to 20 min only resulted in a minor increase in image noise, e.g., for 2i5s and a 4 mm filter, the CV values were 31 ± 8% and 29 ± 9% for 20 and 30 min, respectively. 

**Figure 5:**
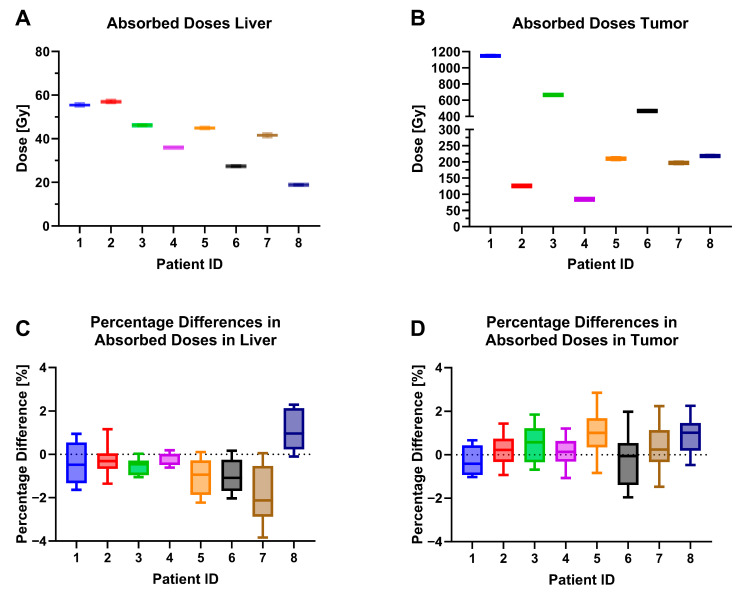
(**A**) Absorbed doses in treated liver background (30 mm spherical VOI) across all reconstruction parameter sets (1i5s-3i5s, 0, 2, 4, 6, mm filters), (**B**) absorbed doses in tumor area across all reconstruction parameter sets, (**C**) percentage differences in the absorbed dose in the liver background across all reconstruction parameter sets to the reference 2i5s, 4 mm filter, 30 min acquisition time, (**D**) percentage differences in absorbed tumor dose across all reconstruction parameters sets.

The results of healthy liver and tumor dosimetry are shown in Figure 5. As expected, the tumor ADs were higher, on average, across reconstructions and patients of 389.6 ± 339.4 Gy than for healthy liver ADs with 45.7 ± 13.5 Gy. The reported ADs in this simplified dosimetry approach are highly dependent on the localization of the single spherical VOIs, due to the heterogeneity of microsphere deposition. However, this approach is still valid for the purpose of this research to compare the impacts of different acquisition and reconstruction parameters on ADs, since the localization of the respective VOIs is identical in all of the investigated sets of images. 

The maximum percentage differences in ADs across the different acquisition times and reconstruction parameters were −3.89% and 3.59% for healthy liver tissue and for tumors, respectively. For detailed information on the calculated mean doses for livers and tumors for each dataset, see Supplementary Materials Tables S2 and S3. 

### 3.4. Patient Study Cases

#### 3.4.1. Patient 3—NET Metastases

The 66-year-old patient was initially diagnosed with an adenocarcinoma of the pancreas pT2 pN0 G2 13 years before radioembolization treatment. In the course of a partial pancreatic resection with splenectomy, gemcitabine was administered as the adjuvant chemotherapy. The hepatic recurrence was treated with hemihepatectomy, and simultaneous concomitant cholecystectomy was performed. The initial histological diagnosis was a neuroendocrine carcinoma (NEC) of the pancreas; consequently, the patient received chemotherapy with carboplatin and etoposid. Subsequently, the patient was referred to our ENETS Center of Excellence, where a G3 neuroendocrine tumor (NET) with a Ki67 index of 25% was diagnosed by the reference pathology. CT imaging showed renewed progression, while somatostatin receptor (SSR) expression categorized as a Krenning score of 4 was identified by PET/CT imaging. Thus, the patient received four cycles of Lu177-PRRT. After two years of stable disease, hepatic oligoprogression indicated radioembolization treatment, as recommended by the interdisciplinary tumor board of our comprehensive cancer center. 

**Figure 6:**
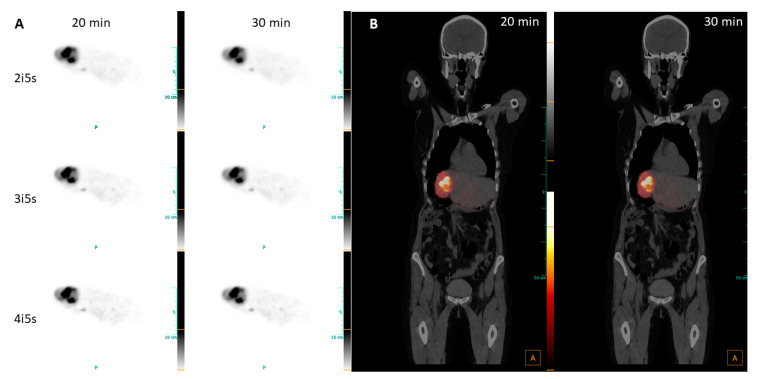
(**A**) Transversal views of PET images of patient 3, diagnosed with NET metastases, reconstructed with 2i5s, 4 mm filter; (**B**) fused coronal PET/CT images, reconstructed with 2i5s, 4 mm filter.

The treatment planning showed a perfused liver volume of 1006 mL, including the tumor with approximately 90 mL in segment VI, as well as multiple lesions. It was decided to treat the patient with 2031 MBq of Y-90 glass spheres in one catheter position to reach a mean absorbed dose in the tumor area of only 100 Gy, in comparison to the 120 Gy standard. The heavily pre-treated liver indicated an activity reduction to ensure liver function after treatment. Thus, the planned absorbed dose to healthy liver parenchyma was limited to 40 Gy. The 30 min post-therapy Y-90 PET scan was performed 18 h p.i. and showed good targeting of the tumor in segment VI. The images clearly show the perfused non-tumorous liver with high noise, which increased with an increasing number of iterations (Figure 6A). No differences in contrast or uptake are visible for the different reconstructions and, in addition, the fused images show no visible differences between the 20 min and 30 min scans (Figure 6B). Although the images appear to be blurry, the image quality is sufficient to identify tumor and non-tumorous liver regions. Moreover, no dystopic Y-90 activity outside the planned treatment volume was detected.

#### 3.4.2. Patient 5—HCC

An 81-year-old patient was diagnosed with a grade 2 HCC in the right liver lobe. After a new progression under three consecutive TACE sections, the interdisciplinary tumor board decided to perform radioembolization. Simulation of sphere distribution with Tc-99m-MAA was performed 6 days prior to therapy. The treatment planning showed 132 mL of tumor volume with a TNR of 4.04. The partition model resulted in a treatment activity of 1330 MBq, leading to a mean dose to the tumor of 135 Gy, while 33 Gy was calculated for the 1259 mL of non-tumorous liver parenchyma of the right lobe. The tumor treatment was carried out via intra-arterial application of 1285 MBq of Y-90 resin spheres via a single catheter position in the right hepatic artery. Post-therapy PET/CT imaging with 30 min acquisition time was performed 42 h p.i.

**Figure 7:**
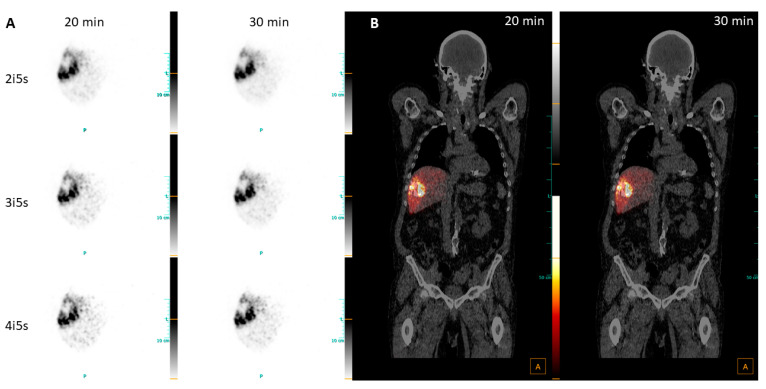
(**A**) Transversal views of PET images of patient 5, diagnosed with HCC, reconstructed with 2i5s; (**B**) fused coronal PET/CT images, reconstructed with 2i5s.

The images clearly visualize the Y-90 deposition within the tumor and spared necrotic tissue in the central area (Figure 7A). In the untreated left liver lobe, no Y-90 uptake is detected. A visual comparison of the PET images with different reconstructions did not reveal significant differences in contrast or visualization of Y-90 uptake, but the expected increase in noise with a rising number of iterations was clearly visible, especially in the treated liver background. More prominent noise for a shorter acquisition time was better visualized in the fused images (Figure 7B), but still not displaying any Y-90 uptake outside the liver.

#### 3.4.3. Patient 8—CRC Metastases

The 50-year-old patient was diagnosed with hepatic metastatic sigmoid carcinoma. After an initial diagnosis, the patient underwent surgery of the primary tumor and multiple chemotherapeutic treatments, including several cycles of FOLFIRI + pantumumab, as well as FOLFOX + bevacizumab, and a modified scheme of folinic acid + 5-fluorouacil and trifluridine/tipiracil. In addition, an SBRT of a single metastasis in liver segment VI was performed 2 years before radioembolization treatment. For treatment planning, Tc-99m-MAA SPECT/CT and diagnostic contrast-enhanced CT were performed the same day, revealing progredient bilobar hepatic dissiminated metastasis with central necrosis. The SPECT/CT showed a total liver volume of 3369 mL, while the perfused liver volume (tumor plus normal liver) was estimated at 2287 mL, as based on Tc-99m-MAA uptake. The tumor volume was calculated to be 908 mL. We decided to exclude necrotic liver areas from dose calculation, as these failed to show Tc99m-MAA uptake in SPECT/CT, and were thus considered to receive no therapeutic spheres under radioembolization. The TNR was calculated to be approximately 3.7 and lung shunt fraction was estimated at 1%. It was decided to treat the patient via one central catheter position with 4659 MBq of Y-90 glass spheres to achieve a planned absorbed mean dose of 100 Gy. The planned dose was reduced from the standard 120 Gy with respect to the heavily pretreated liver and high tumor load. The AD for the untreated non-tumorous liver parenchyma was limited to 25 Gy.

**Figure 8:**
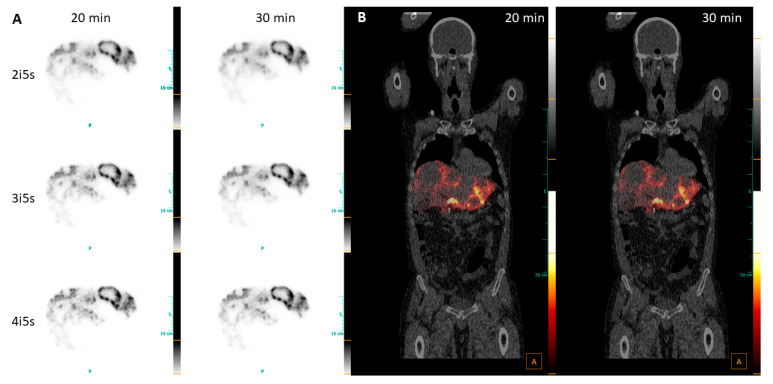
(**A**) Transversal views of PET images of patient 8, diagnosed with CRC reconstructed with 2i5s, 4 mm filter; (**B**) fused coronal PET/CT images, reconstructed with 2i5s, 4 mm filter.

The post-therapeutic Y-90 PET imaging was performed 22 h p.i. with 30 min acquisition time. The relatively high amount of radioactivity compared to treatment with resin spheres led to higher contrast, clearly visualizing moderate uptake in the perfused liver background and high uptake in the tumor periphery, in contrast to the necrotic area without uptake at all (Figure 8A).

The visual impressions of the different numbers of iterations and different acquisition times show negligible differences. The fused PET and CT images support the impression and reveal no significant differences in visualizing the Y-90 accumulation between 30 min and 20 min acquisition times (Figure 8B).

## 4. Discussion

This study determined the optimum scan protocol and image reconstruction settings for post-therapy imaging of radioembolization patients with the Biograph Vison Quadra LAFOV PET/CT scanner. For the evaluation, the impacts of the scan duration, sensitivity mode, number of iterations for the image reconstruction and image filter size on the contrast recovery, image noise, IQ and accuracy for dosimetry were assessed.

### 4.1. IEC Phantom as Comparison Benchmark

The IEC phantom study showed that the noise increased with shorter scan durations, but the contrast recovery was largely preserved for the 20 min and 30 min scans compared to a 24 h reference. These results are consistent with those reported by Zeimpekis et al. [9]. On average, the CRC values in their research were higher compared to our findings, e.g., for 30 min 34% vs. 23% (10 mm sphere), 45% vs. 27% (13 mm), 57% vs. 62% (17 mm), 62% vs. 65% (22 mm), 72% vs. 68% (28 mm) and 85% vs. 75% (37 mm). This can be explained by the higher SBR of 10:1 compared to the SBR of 4:1 used in our study. 

The reason for elaborating an SBR of 4:1 was that the TNR in terms of activity concentration is often less than 10:1 in patients undergoing Y-90 radioembolization, especially for tumor entities other than HCC [4,25].

The lung residual error in our research (12%) was lower than that reported by Zeimpekis et al. (16%), which can be explained by the higher background activity concentration of 270 kBq/mL in our study compared to 100 kBq/mL, as the overestimation of the lung shunt fraction is increased at lower activities [13].

Soderlund et al. [14] performed a comparable experiment using a Y-90-filled IEC phantom with an SBR of 4:1 in the Biograph mCT (Siemens Healthineers, Knoxville, TN, USA) SAFOV PET/CT scanner. Despite the long acquisition time of 12 h, the contrast recovery was largely reduced compared to our findings, with CRC values of <20% vs. 23% (10 mm sphere), <50% vs. 62% (17 mm) and <60% vs. 75% (37 mm sphere), and a higher lung residual error of >15% (our study: 12%) was reported. 

For the SAFOV Biograph Vision PET/CT scanner, which uses the same detector technology as the Biograph Vision Quadra, the optimal image reconstruction for Y-90 imaging in radioembolization was evaluated by Kunnen et al. [13]. They recommended image reconstruction with 3i5s and no post-reconstruction filtering, reporting CRC values of approximately 50% (10 mm sphere) to 90% (37 mm) in an IEC phantom with SBR 8:1 and 30 min acquisition time. 

Due to its use within the NEMA-NU 2018 standard, the IEC phantom is well suited for comparing the performance of different scanners, but is not specifically designed to assess PET image quality and quantification for scans of radioembolization patients. For this reason, additional evaluations were performed using the AbdoMan phantom, focusing on the liver as the relevant organ for this therapy.

### 4.2. Scan Protocol and Image Reconstruction Parameter Optimization with the AbdoMan Phantom

The results of the AbdoMan phantom evaluations showed that the use of the UHS mode largely improved the image noise compared to the HS mode (e.g., for 30 min from the CV values were 40% (HS) to 29% (UHS)). The noise improvement of factor 1.4 can be explained by the larger acceptance angle used in UHS, which results in an increased sensitivity. The results are in accordance with a noise improvement of up to a factor of 1.49 in the center FOV for the UHS mode, as reported by Schmidt et al. [26] for F-18 phantom evaluations in the Biograph Vision Quadra. As the liver is located close to the center FOV in post-radioembolization scans, these scans benefit from the increased sensitivity of the UHS mode in this area. 

In terms of the number of iterations for reconstruction, 2i5s was found to be optimal for Y-90 imaging on the Biograph Vision Quadra, providing a reasonable trade-off between image noise and contrast recovery. Of note, with 2i5s for Y-90 imaging with the Biograph Vision Quadra, the number of iterations is much lower than the 4i5s typically used for other isotopes with a higher branching ratio such as F-18 [18], Ga-68 or Zr-89 [27]. Zeimpekis et al. [9] determined the highest SNR for reconstructions with 2i5s and a 2 mm FWHM Gaussian filter for the IEC phantom, while lesion detectability, which can be related to contrast recovery, was best with 4i5s. In our research, 4 mm was found to be the optimum filter size, as it maintained contrast recovery and sufficiently mitigated noise. Larger filter sizes up to 8 mm did not further reduce image noise.

Due to the low event statistics of Y-90, the filter size was larger than the 0–2 mm typically used for F-18-based tracer examinations on the Biograph Vision Quadra [28,29]. However, larger filter sizes could also be considered for isotopes with a high branching ratio if shorter scan times are required, e.g., a 6 mm filter for F-18-based tracers and a scan time of 2 min to maintain noise <15% for an axial extent of 103 cm [30] or 7 mm and 5 mm to meet the EARL 1 and EARL 2 standard specifications, respectively [31].

Larger filter sizes are associated with a potential loss in the detection of small structures, which is more acceptable for post-therapeutic Y-90 imaging than for other diagnostic scans. The reason for this is that for post-therapeutic dosimetry, the accurate quantification of activity with precise localization in predefined regions is more important than lesion detectability [32,33].

In addition, a larger filter reduces the image noise in the liver background, which helps to accurately determine the activity concentration and absorbed dose. This region is of uttermost importance, as the main constraint for radioembolization therapy is to keep the dose for healthy liver parenchyma reasonably low.

### 4.3. Impacts of Scan Protocol and Reconstruction Parameters on Post-Therapeutic Dosimetry

The results obtained from the analysis of the patient cohort were consistent with the results obtained from the phantom measurements. The analysis of the CV in the liver background showed an improvement in the CV for a filter size of up to 4 mm, e.g., for 2i5s and 30 min, from 38% (0 mm filter) to 31% (4 mm). Consistent with the phantom study, a further increase in the filter size also did not result in a further reduction in image noise for the patient data.

Only negligible percentage differences in the calculated AD values were found across all of the reconstruction parameter sets (maximum deviation from reference −3.89%). This demonstrates that all reconstruction parameter sets can be used for dosimetry regions with low but homogeneous uptake, such as for healthy liver tissue, as well as for higher and potentially heterogeneous uptake, such as for tumor tissue. In conclusion, the optimal trade-off between the CV and absorbed dose quantification was found to be 2i5s in combination with a 4 mm FWHM Gaussian filter.

Of note, neither image noise nor quantification was notably degraded for the 20 min scan, indicating that the high sensitivity of the Biograph Vision Quadra allows for such short scans. 

The results obtained from the patient cases demonstrated high image quality and clear visualization of tumor tissues for different types of disease with different vascularization and dissemination, resulting in different activity distributions. 

For the patient cases, the 20 min scans compared to the 30 min scans revealed slightly increased noise, but did not notably affect visualization of the lesions. In conclusion, together with the above results, this confirms that short scan times of 20 min to 30 min for post-therapeutic Y-90 PET imaging with the Biograph Vision Quadra are suitable for post-therapeutic radioembolization dosimetry, and can help to bring this into clinical routine.

### 4.4. Limitations in the Simplified S-Value-Based Dosimetry and Considerations for Advanced Dosimetric Approaches

It should be noted that the approach used for dosimetry in this study is a simplified approach, and therefore a partial volume effect correction should be considered to obtain more accurate absolute dose values. However, in this study, the same VOI size was used for all of the patient evaluations, so this correction was not necessary for the comparison of the AD values. In addition, the VOIs were not placed in the physiological tumor region nor the healthy liver region, but in the visually determined tumor-treated volume and non-tumorous liver tissue perfused with activity. Therefore, the absolute dose values did not fully represent the actual tumor or liver dose. However, this simplified approach was possible to use in this research to evaluate the impact of different reconstruction parameters and acquisition times. It remains subject to future investigations to determine how the parameters evaluated in this study affect voxel-based dosimetry, such as for 3D-absorbed dose maps obtained by dose kernel convolution [34] orindividual patient Monte Carlo simulations [35]. More importantly, whether the parameters will impact voxelized 3D dose maps to such an extent they influence clinical decision-making with regard to potential re-treatment after revealing under-treated tumors or tumor regions. Nevertheless, the optimized protocol and image reconstruction parameters provide a basis for establishing post-therapeutic Y-90 radioembolization imaging in clinical practice, enabling routine post-treatment dosimetry and further investigation of treatment success based on absorbed doses.

## 5. Conclusions

In this study, we evaluated different image reconstruction parameters, acquisition times and filter sizes for Y-90 PET imaging after radioembolization with the Biograph Vision Quadra LAFOV PET/CT scanner. Based on two phantom studies and a study involving patients with different treatments and diseases, we determined that the optimum protocol for accurate post-therapeutic dosimetry is to use 2i5s with a 4 mm filter in combination with scan times as short as 20 min.

## Figures and Tables

**Table 1:** Characteristics of the patients involved in the study.

Patient ID	Age	Sex	Injected Activity [MBq]	Sphere Type	Treated Liver Volume [mL]	Tumor Volume [mL]	Tumor Entity	Prior Treatment
1	80 y	Male	1314	Resin	477	n.a. *	HCC	-
			1269	Resin		n.a. *		
2	79 y	Male	1456	Resin	1310	537	NET	Lu177-PRRT, SSA
3	66 y	Female	2031	Glass	1006	90	NET	surgery, gemicitabine, etoposide/cisplatin, Lu-177 PRRT + temozolomide + capecitabine, SSA
4	76 y	Male	1270	Resin	1461	170	CRC	FOLFOX, FOLFIRI + ramicirumab, pembrolizumab
			442	Resin	226	127		
5	81 y	Male	1285	Resin	1392	132	HCC	3 × TACE
6	46 y	Male	1411	Glass	554	96	NET	surgery, Lu177-PRRT, SSA, 2 × TACE
			2578	Glass	1060	76		
7	61 y	Male	3550	Glass	1891	n.a. *	CRC	FOLFOX + bevacizumab, surgery, partial ALPPS
8	40 y	Male	4659	Glass	2287	908	CRC	FOLFIRI + pantumumab, FU/FOL + bevacizumab, SBRT, FOLFOX + bevacizumab, trifluridine/tipiracil

* Not applicable because of intended radiation segmentectomy or lobectomy. Lu177-PRRT = lutetium-177-peptide receptor radiotherapy, SSA = somatostatin analogues, FOLFOX = folinic acid + 5-fluorouracil + oxaliplatin, FOLFIRI = folinic acid + 5-fluorouracil + irinotecan, TACE = transarterial chemoembolization, FU/FOL = 5-fluorouracil + folinic acid, ALPSS = associating liver partition with portal vein ligation for staged hepatectomy, SBRT = stereotactic body radiation therapy.

**Table 2:** Contrast recovery coefficients for different spheres of the IEC phantom, scan times and filter sizes.

Scan Time	Filter FWHM [mm]	Contrast Recovery Coefficient for Different Spheres [%]					
		10 mm	13 mm	17 mm	22 mm	28 mm	37 mm
24 h	0	24	35	52	63	65	76
	2	23	34	51	62	64	75
30 min	0	24	29	64	66	69	76
	2	23	27	62	65	68	75
20 min	0	20	35	67	70	72	77
	2	20	34	66	69	71	77

**Table 3:** CVs in the patient liver background for different scan durations, number of iterations and filter sizes.

		Mean CV [%] and Standard Deviation		
Scan Time [min]	Filter FWHM [mm]	Iterations		
		2i5s	3i5s	4i5s
30	0	34 ± 8	43 ± 9	51 ± 11
	2	32 ± 8	31 ± 8	45 ± 11
	4	29 ± 9	34 ± 10	39 ± 11
	6	29 ± 9	34 ± 10	38 ± 11
20	0	38 ± 7	49 ± 8	58 ± 11
	2	34 ± 7	41 ± 8	51 ± 10
	4	31 ± 8	38 ± 8	43 ± 10
	6	31 ± 8	37 ± 8	42 ± 10

## Data Availability

The phantom data presented in this study are available on reasonable request from the corresponding author. Patient data cannot be made publicly available for ethical and legal reasons, as public availability would compromise patient confidentiality.

## Supplementary Materials

The following supporting information can be downloaded at: https://www.mdpi.com/article/10.3390/diagnostics13223418/s1, Table S1: CRC values for the AbdoMan phantom for 0, 2, 4, 6, 8 mm filter and acquisition time of 30 min; Table S2: Mean absorbed doses in liver VOIs (30 mm sphere) for every patient for all studied reconstruction parameter sets (2i5s–4i5s, 20 and 30 min, filter sizes 0, 2, 4, 6 mm); Table S3: Mean absorbed doses in tumor VOIs (30 mm sphere) for every patient for all studied reconstruction parameter sets (2i5s–4i5s, 20 and 30 min, filter sizes 0, 2, 4, 6 mm).
