# Supplementary material for: Optimization of Y-90 Radioembolization Imaging for Post-Treatment Dosimetry on a Long Axial Field-of-View PET/CT Scanner
Source: Diagnostics (Basel). 2023 Nov 9;13(22):3418. doi: 10.3390/diagnostics13223418 (PMC10670048; doi:10.3390/diagnostics13223418)
Supplement: Supplementary file 1 [file diagnostics-13-03418-s001.zip › diagnostics-2671130-supplementary.pdf]

diagnostics

MDPI

Supplementary Table S1. CRC values for the AbdoMan phantom for 0, 2, 4, 6, 8 mm filter and acquisition time of 30 min.

	Contrast Recovery Coefficients			
**Filter FWHM [mm]**	**Sphere Size [mm]**			
	**50**	**40**	**30**	**20**
**0**	73	64	59	54
**2**	73	64	58	52
**4**	72	63	57	51
**6**	72	63	57	51
**8**	72	63	57	51

Supplementary Table S2. Mean absorbed doses in liver VOIs (30 mm sphere) for every patient for all studied reconstruction parameter sets (2i5s-4i5s, 20 and 30 min, filter sizes 0, 2, 4, 6 mm).

			Mean Absorbed Dose in Liver VOI [Gy]							
**Acquisition Time [min]**	**Filter FWHM [mm]**	**Iterations**	**ID 1**	**ID 2**	**ID 3**	**ID 4**	**ID 5**	**ID 6**	**ID 7**	**ID 8**
30	**0**	**2i5s**	55.7	57.2	46.4	36.0	45.3	27.6	42.4	18.6
	**0**	**3i5s**	56.2	57.2	46.3	35.8	45.1	27.1	41.6	18.7
	**0**	**4i5s**	56.0	57.8	46.3	35.8	45.1	27.4	41.1	18.6
	**2**	**2i5s**	55.7	57.1	46.4	36.0	45.4	27.6	42.4	18.6
	**2**	**3i5s**	56.1	57.0	46.3	35.8	45.2	27.2	41.6	18.7
	**2**	**4i5s**	56.0	57.7	46.3	35.8	45.2	27.4	41.1	18.7
	**4**	**2i5s**	55.6	57.0	46.4	36.0	45.4	27.7	42.4	18.7
	**4**	**3i5s**	56.1	56.9	46.3	35.8	45.2	27.2	41.7	18.8
	**4**	**4i5s**	55.9	57.5	46.3	35.8	45.3	27.4	41.2	18.7
	**6**	**2i5s**	55.6	57.0	46.4	36.0	45.4	27.7	42.4	18.7
	**6**	**3i5s**	56.0	56.8	46.3	35.8	45.2	27.2	41.7	18.8
	**6**	**4i5s**	55.9	57.4	46.3	35.8	45.2	27.4	41.2	18.7
20	**0**	**2i5s**	55.0	56.6	46.2	36.0	44.6	27.5	42.1	18.9
	**0**	**3i5s**	54.8	56.9	46.0	36.0	44.4	27.1	41.3	19.0
	**0**	**4i5s**	55.3	57.1	45.9	36.0	44.4	27.3	40.7	19.0
	**2**	**2i5s**	55.0	56.5	46.2	36.0	44.7	27.5	42.1	18.9
	**2**	**3i5s**	54.8	56.8	46.0	36.0	44.4	27.1	41.3	19.0
	**2**	**4i5s**	55.2	57.0	46.0	36.0	44.4	27.3	40.8	19.0
	**4**	**2i5s**	54.9	56.4	46.2	36.0	44.7	27.6	42.2	18.9
	**4**	**3i5s**	54.8	56.7	46.0	36.0	44.5	27.1	41.4	19.0
	**4**	**4i5s**	55.2	56.9	46.0	36.0	44.5	27.3	40.9	19.1
	**6**	**2i5s**	54.9	56.4	46.2	36.0	44.8	27.6	42.2	18.9
	**6**	**3i5s**	54.8	56.7	46.0	36.0	44.5	27.1	41.4	19.0
	**6**	**4i5s**	55.2	56.9	46.0	36.0	44.5	27.3	40.9	19.1

Supplementary Table S3. Mean absorbed doses in tumor VOIs (30 mm sphere) for every patient for all studied reconstruction parameter sets (2i5s-4i5s, 20 and 30 min, filter sizes 0, 2, 4, 6 mm)

			Mean Absorbed Dose in Tumor VOI [Gy]							
**Acquisition Time [min]**	**Filter FWHM [mm]**	**Iterations**	**ID 1**	**ID 2**	**ID 3**	**ID 4**	**ID 5**	**ID 6**	**ID 7**	**ID 8**
30	**0**	**2i5s**	1156.9	126.2	664.6	84.7	209.1	470.2	197.5	217.2
	**0**	**3i5s**	1156.5	125.9	665.2	85.4	211.7	464.3	199.9	219.6
	**0**	**4i5s**	1157.0	126.4	664.7	85.6	212.4	462.0	200.7	220.5
	**2**	**2i5s**	1150.0	125.6	661.9	84.6	208.1	469.5	196.3	216.6
	**2**	**3i5s**	1149.6	125.3	662.5	85.2	210.5	463.7	198.6	219.0
	**2**	**4i5s**	1149.9	125.9	662.1	85.4	211.3	461.3	199.5	219.8
	**4**	**2i5s**	1140.6	124.8	658.1	84.3	206.6	468.5	194.8	215.7
	**4**	**3i5s**	1140.1	124.6	658.8	85.0	209.0	462.8	197.0	218.1
	**4**	**4i5s**	1140.4	125.1	658.4	85.2	209.7	460.5	197.8	218.9
	**6**	**2i5s**	1138.8	124.6	657.3	84.3	206.3	468.3	194.4	215.5
	**6**	**3i5s**	1138.3	124.4	658.1	84.9	208.7	462.6	196.7	217.9
	**6**	**4i5s**	1138.5	125.0	657.7	85.1	209.4	460.3	197.4	218.7
20	**0**	**2i5s**	1156.7	127.4	673.5	84.2	210.6	478.8	196.5	217.5
	**0**	**3i5s**	1157.7	127.1	674.1	84.9	213.2	473.1	198.8	220.4
	**0**	**4i5s**	1157.7	127.3	673.6	85.1	214.0	470.7	199.7	221.5
	**2**	**2i5s**	1149.8	126.8	670.8	84.0	209.4	477.9	195.3	216.9
	**2**	**3i5s**	1150.6	126.5	671.4	84.6	212.0	472.3	197.6	219.7
	**2**	**4i5s**	1150.6	126.8	671.0	84.9	212.8	470.0	198.4	220.7
	**4**	**2i5s**	1140.3	125.9	667.0	83.7	207.9	476.7	193.8	216.0
	**4**	**3i5s**	1140.9	125.7	667.7	84.3	210.3	471.2	195.9	218.8
	**4**	**4i5s**	1140.9	126.0	667.3	84.5	211.1	468.9	196.6	219.7
	**6**	**2i5s**	1138.5	125.8	666.2	83.7	207.6	476.5	193.5	215.8
	**6**	**3i5s**	1139.0	125.6	666.9	84.3	210.0	471.0	195.6	218.6
	**6**	**4i5s**	1139.0	125.9	666.6	84.5	210.7	468.7	196.3	219.5
